# Transcriptomic profiling reveals macrophage gene signatures associated with lactylation-related pathways in chronic obstructive pulmonary disease

**DOI:** 10.3389/fgene.2025.1653163

**Published:** 2025-10-13

**Authors:** Hui Guo, Weilin Sun, Fang Zhao, Yang Yu, Xiaoyun Zhao, Daqiang Sun

**Affiliations:** ^1^ Department of Respiratory and Critical Care Medicine, Tianjin Chest Hospital, Tianjin University, Tianjin, China; ^2^ Tianjin Medical University Cancer Institute & Hospital National Clinical Research Center for Cancer, Tianjin, China

**Keywords:** chronic obstructive pulmonary disease, macrophage, lactylation, biomarkers, experimental verification

## Abstract

**Background:**

Macrophages contribute to the immune dysregulation observed in chronic obstructive pulmonary disease (COPD). Additionally, lactylation exerts an indirect influence on COPD pathogenesis. However, the specific biomarkers linked to macrophage activation in COPD and the underlying molecular mechanisms remain poorly understood. This study aimed to identify these biomarkers and elucidate the associated molecular pathways.

**Methods:**

Data were retrieved from public databases. A comprehensive analysis was conducted using weighted gene co-expression network analysis (WGCNA), immune infiltration analysis, differential expression analysis, correlation studies, machine learning, receiver operating characteristic (ROC) analysis, and expression level validation to identify macrophage lactylation-related biomarkers in COPD. The nomogram model, Gene Set Enrichment Analysis (GSEA), molecular regulatory networks, compound predictions, and molecular docking were employed to further explore the roles of these biomarkers in COPD. Clinical samples were used to validate the expression levels of the identified biomarkers.

**Results:**

Three key biomarkers—ALDH2, ASGR2, and CYP1B1—were identified. The nomogram model based on these biomarkers accurately predicted the mortality of patients with COPD. GSEA suggested that the biomarkers are likely involved in metabolic pathways and B-cell receptor signaling. Five transcription factors (TFs), including STAT3, were associated with all identified biomarkers. Eight compounds, including bisphenol A, were linked to multiple biomarkers, with CYP1B1 exhibiting the strongest binding affinity to benzo(a)pyrene. *In vitro* experiments confirmed the validity of the bioinformatics findings.

**Conclusion:**

This study identified three biomarkers, offering new perspectives on potential therapeutic targets for COPD.

## 1 Introduction

Chronic obstructive pulmonary disease (COPD) is a heterogeneous respiratory disorder characterized by concurrent injury and remodeling of the airways, lung parenchyma, and pulmonary vasculature. These lung impairments lead to progressively worsening airflow limitation, which in turn results in increased dyspnea, functional disability, and premature death (Qian et al., 2023). COPD is a major global health burden, ranking as the fourth leading cause of death worldwide, characterized by high prevalence, high mortality, and significant healthcare resource consumption (Wen et al., 2018; Ferrera et al., 2021). It showed an annual incidence rate of roughly 12%, with a global prevalence ranging from 9% to 10% among people aged 40 and older (Adeloye et al., 2015; Diseases and Injuries, 2020). The development of COPD involves a complex interplay of genetic susceptibility (e.g., α1-antitrypsin deficiency), environmental exposures (e.g., smoking, air pollution), and dysregulated immune responses (e.g., neutrophil/macrophage activation, protease-antiprotease imbalance). Dysregulation of immune responses in COPD leads to the activation of multiple immune cells, such as neutrophils, macrophages, lymphocytes, and eosinophils (Rabe et al., 2023). These cells secrete a large number of inflammatory mediators and cytokines, including interleukin-6 (IL-6), interleukin-8 (IL-8), tumor necrosis factor-α (TNF-α), and chemokines. They can recruit more immune cells to the lungs, perpetuate the inflammatory process, and contribute to airway remodeling and lung tissue destruction. However, the complex pathogenesis of COPD remains incompletely understood (Zhu et al., 2025). Early diagnosis and timely therapeutic intervention are critical in improving prognosis and survival rates for individuals with COPD (Lin et al., 2023). Current diagnostic methods for COPD mainly include pulmonary function tests, chest X-ray radiography, and computed tomography (CT), etc. Due to the limited sensitivity and specificity of the equipment, there are certain difficulties in distinguishing COPD from other lung diseases. Moreover, these methods have poor visualization of mild lesions and suboptimal performance in the differential diagnosis of early-stage COPD (Kumar et al., 2024; Chen et al., 2025). In addition, pharmacological treatments currently focus on antibiotics and corticosteroids to manage inflammation, yet no clinically effective targeted therapies are available. Therefore, understanding the underlying molecular mechanisms and identifying new biomarkers are essential for establishing early diagnostic standards and discovering precision therapeutic targets for COPD.

Lactylation, a novel post-translational modification that involves the covalent attachment of lactate to lysine residues, has emerged as a critical regulatory mechanism in various cellular processes (Zhang et al., 2019; Gaffney et al., 2020). Recent studies indicate that lactylation plays a pivotal role in diverse physiological and pathological processes, including immunity (Chen et al., 2022), metabolism (Chen et al., 2021; Merkuri et al., 2024), and cancer (Yu et al., 2021), by modifying both histones and non-histone proteins. A recent study demonstrated that lactylation significantly influences the phenotype and functional properties of immune cells, including macrophage polarization (Irizarry-Caro et al., 2020) and T cell reprogramming (Lopez Krol et al., 2022). Pulmonary macrophages, encompassing tissue-resident macrophages (TRMs) and monocyte-derived macrophages (Hashimoto et al., 2013; Byrne et al., 2016), play pivotal roles in COPD pathophysiology. The M1 and M2 phenotypic states of these macrophages mediate distinct functional outcomes (Kim et al., 2024). Notably, lactylation has been identified as a key modulator of macrophage polarization, influencing both inflammatory mediator production and tissue repair capacity (Irizarry-Caro et al., 2020). Moreover, lactylation regulates gene expression related to inflammation, cytokine synthesis, and metabolic pathways in macrophages, ultimately affecting their activation states and functional outcomes (Cui et al., 2021; Wang et al., 2022; Li et al., 2024). Abnormal macrophage function, immune dysregulation, and sustained inflammation are well-established contributors to COPD pathogenesis (Lee et al., 2021). Therefore, investigating the role of lactylation in macrophage activation is essential for advancing our understanding of COPD pathogenesis and for developing novel therapeutic approaches and molecular targets.

This study utilized two transcriptomic datasets comprising blood samples from smokers with COPD and smokers with normal lung function. Differentially expressed genes (DEGs) related to macrophage lactylation between COPD and control samples were identified through various bioinformatics approaches, including differential expression analysis, immune infiltration analysis, and weighted gene co-expression network analysis (WGCNA). The underlying mechanisms of these biomarkers in COPD were further explored using a nomogram model, Gene Set Enrichment Analysis (GSEA), molecular regulatory networks, compound prediction, and molecular docking. Additionally, clinical samples were analyzed to validate the expression levels of the identified biomarkers, confirming the bioinformatics findings. In summary, this study provides a solid foundation for a deeper understanding of the molecular mechanisms driving COPD and offers potential avenues for novel therapeutic strategies.

## 2 Materials and methods

### 2.1 Data collection

Transcriptome data (GSE100153, GSE124180) for COPD were sourced from the GEO database (https://www.ncbi.nlm.nih.gov/geo/). Based on the sample size and homogeneity, use GSE100153 as the training set and GSE124180 as the validation set. The training set GSE100153 based on the GPL6884 platform (Illumina HumanWG-6 v3.0 Expression BeadChip) included 19 COPD and 24 control whole blood samples, while the validation set GSE124180 based on the GPL16791 platform (Illumina HiSeq 2000 RNA Sequencing) comprised 6 COPD and 15 control whole blood samples. Lactylation-related genes (LRGs) were identified, including a lactylation enzyme, EP300, and 6 delactylases (HDAC1-3, SIRT1-3), as well as 327 lactylated proteins documented in the literature (Cheng et al., 2023). Finally, 332 LRGs were acquired (Supplementary Table S1).

### 2.2 Differential expression analysis

For the GSE100153 dataset, differential expression analysis was performed using the “limma” package (v 3.54.0) (Ritchie et al., 2015), identifying DEGs between COPD and control samples (COPD vs. control) with |log_2_ fold change (FC)| > 0.5 and P < 0.05. Volcano plots were generated using the “ggplot2” package (v 3.4.1) (Gustavsson et al., 2022) to visualize all DEGs, with the top 5 up- and downregulated DEGs labeled. Additionally, a heatmap displaying the expression levels of the top 25 up- and downregulated DEGs was created using the “pheatmap” package (v 1.0.12) (Gu and Hubschmann, 2022).

### 2.3 Immune infiltration

To investigate immune cell variations in COPD development, the xCell algorithm was applied to assess the infiltration levels of 34 immune cell categories in the GSE100153 dataset (Aran et al., 2017). The Wilcoxon test was used to compare immune cell infiltration between COPD and control groups, and differential immune cells were identified (P < 0.05). Results were visualized using the “ggplot2” package (v 3.4.1).

### 2.4 Acquisition of macrophage-related module genes

Focusing on immune cells with significant differences between COPD and control groups and those associated with macrophages, WGCNA was conducted to identify module genes linked to macrophages in the GSE100153 dataset using the “WGCNA” package (v 1.72) (Langfelder and Horvath, 2008). Initially, the “GoodSamplesGenes” function was employed to cluster samples and remove outliers, with a height threshold of 70 for the clustering tree. The optimal soft threshold (power) above the red cut line was determined by setting R^2^ = 0.8. Based on the selected soft threshold, genes were classified into multiple modules. The minModuleSize was set to 30, and the module merging parameter (mergeCutHeight) was 0.25. Key modules were identified by correlating modules with phenotypic traits, and a correlation heatmap was generated using the “pheatmap” package (v 1.0.12) (|cor| > 0.3, P < 0.05). Genes within the key modules were classified as macrophage-related genes (MRGs), reflecting their association with macrophage-related phenotypic traits.

### 2.5 Identification and function of candidate genes

To identify genes associated with macrophage lactylation, the “cor” function was used to examine the correlation between LRGs and MRGs (|cor| > 0.3, P < 0.05) (Xie et al., 2024; Li Y. M. et al., 2025). Genes with significant correlations were considered macrophage LRGs (MLRGs). These MLRGs were then intersected with DEGs to obtain candidate genes, using the “ggvenn” package (v 0.1.9) (Mao et al., 2022). Enrichment analyses for Gene Ontology (GO) functions and Kyoto Encyclopedia of Genes and Genomes (KEGG) pathways were performed on the candidate genes, with significance set at P < 0.05, using the “clusterProfiler” package (v 4.2.2) (Wu et al., 2021). The top 10 most significant GO functions and all relevant KEGG pathways were presented based on P values.

### 2.6 Identification of biomarkers

To identify potential biomarkers, further analyses were conducted on the candidate genes. First, in the GSE100153 dataset, the “Boruta” package (v 8.0.0) (Zhou et al., 2023) was used to apply the Boruta algorithm (7x cross-validation) to identify important genes among the candidate genes. The “e1071” package (v 1.7-13) (Yang et al., 2022) was then used to implement the SVM-RFE algorithm (with 10x cross-validation) to identify key genes at the lowest error rate. The genes identified by both algorithms were intersected using the “ggvenn” package (v 0.1.9). The intersecting genes were then subjected to receiver operating characteristic (ROC) analysis. In both the GSE100153 and GSE124180 datasets, the “pROC” package (v 1.18.0) (Robin et al., 2011) was used to perform ROC analysis, and the area under the curve (AUC) values were calculated. Genes with an AUC >0.7 in both datasets were selected for expression level analysis. The Wilcoxon test was applied in both datasets to compare gene expressions between COPD and control samples (P < 0.05). Genes that exhibited significant expression differences between the COPD and control groups with consistent trends across the two datasets were defined as biomarkers.

### 2.7 Construction of nomogram model

To assess the role of biomarkers in COPD, the “rms” package (v 6.7-0) (Xu et al., 2023) was used to construct a nomogram model based on the identified biomarkers. Each biomarker was assigned a score, and the total score was derived from the sum of the individual scores. Patients with COPD exhibiting higher total scores showed increased mortality rates. The correctness and reliability of the nomogram model were assessed using the ROC curve (generated with the “pROC” package, v 1.18.0) and the decision curve analysis (using the “rmda” package, v 1.0.2) (Kerr et al., 2016).

### 2.8 Gene set enrichment analysis (GSEA)

To investigate the pathways enriched by the biomarkers, the GSE100153 samples were divided into low- and high-expression groups based on the average expression of the biomarkers. The “limma” package (v 3.54.0) was used to calculate log_2_FC values between the two groups. The calculated log_2_FC values were then ranked from largest to smallest. GSEA was performed using the “clusterProfiler” package (v 4.2.2) with parameters P < 0.05 and |NES| > 1. The reference gene collection was “c2.cp.kegg.v7.0.symbols.gmt” from the MSigDB ((https://www.gsea-msigdb.org/gsea/msigdb). The top 5 significantly enriched pathways were displayed.

### 2.9 Prediction of transcription factors (TFs) and compounds

To further investigate the potential molecular regulatory mechanisms of the identified biomarkers, the KnockTF2.0 database (https://bio.liclab.net/KnockTFv2/search.php) was utilized to predict TFs that target these biomarkers. Additionally, the CTD database (https://ctdbase.org/) was employed to identify compound-biomarker interaction pairs and determine potential therapeutic compounds targeting the biomarkers. The regulatory networks between biomarkers and TFs, as well as between biomarkers and compounds, were visualized using Cytoscape software (v 3.8.2) (Shannon et al., 2003).

### 2.10 Molecular docking

To explore the binding capacity between biomarkers and compounds, molecular docking studies were conducted with compounds targeting multiple biomarkers simultaneously. The 3D structures of the compounds were obtained from the PubChem database (https://pubchem.ncbi.nlm.nih.gov/), while the 3D structures of the biomarkers were retrieved from the UniProt database (https://ctdbase.org/) and downloaded from the PDB database (https://www.rcsb.org/). All files were preprocessed using the QuickPrep module of the molecular operating environment (MOE) software (v 2022.02) (Dong et al., 2024). Molecular docking was performed using the Dock module of MOE software (v 2022.02). The molecular docking results were then presented.

### 2.11 Clinical sample verification

To further validate the expression levels of biomarkers between COPD and control samples and confirm the bioinformatics analysis results, RT-qPCR was conducted. Ten frozen whole blood samples (5 COPD and 5 control samples) were collected at Tianjin Chest Hospital. All participants provided informed consent, and the study received ethical approval from the Ethics Review Committee of Approval No. 2025LW-16. Total RNA was extracted from the samples using TRIzol reagent (Vazyme, Nanjing, Jiangsu, China). RNA concentrations were measured using a NanoPhotometer N50. Subsequently, mRNA was reverse transcribed into cDNA using a commercial kit (Yeasen, Shanghai, China), and RT-qPCR was performed under the conditions outlined in Supplementary Table S2. The relative expression of biomarkers was calculated using the 2^−ΔΔCt^ method, with GAPDH as the reference gene. The results were analyzed using GraphPad Prism (v 10.0) (Guo et al., 2022). The expression differences between COPD and control samples were compared using a t-test (P < 0.05).

### 2.12 Statistical analysis

All statistical analyses were performed using R software (v 4.2.2). Wilcoxon tests and t-tests were applied to compare differences between the two groups, with significance set at P < 0.05.

## 3 Results

### 3.1 Identification of MRGs

**FIGURE 1:**
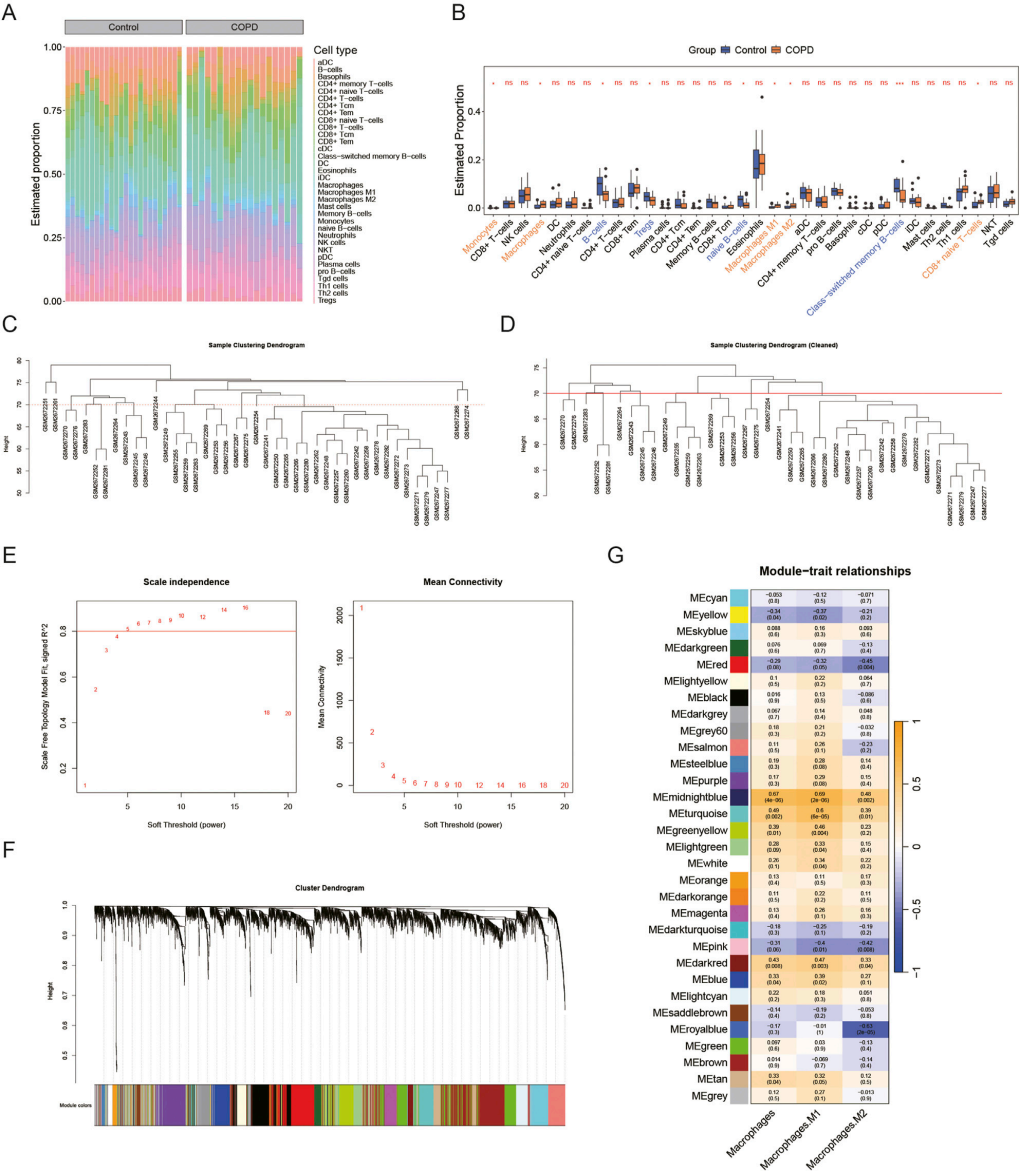
Identification of MRGs. In the GSE100153 dataset, **(A,B)** Immune infiltration analysis of 34 immune cell types (control vs. COPD) is shown in the heatmap **(A)** (Yellow represents high expression, blue-green represents low expression) and box plot **(B)**, ns p > 0.05, *p < 0.05. **(C–G)** Macrophages, M1 macrophages, and M2 macrophages were used as phenotypic traits for WGCNA. All samples are shown in **(C)**, and outlier samples were removed in **(D)**. The appropriate soft threshold (R^2^ = 0.8, power = 5) was selected in **(E)**. The scale-free network construction yielded 30 gene modules **(F)**. Heatmap in **(G)** shows that all three modules are related to phenotypic traits (cor >0.3, P < 0.05).

Using the xCell algorithm, the infiltration levels of 34 immune cell types between the COPD and control groups were assessed (Figure 1A). Among these, the infiltration levels of 9 immune cell types showed significant differences between the two groups (P < 0.05) (Figure 1B). Specifically, three types of macrophages—macrophages, M1 macrophages, and M2 macrophages—demonstrated marked distinctions between COPD and control samples. Given the critical role of macrophages in COPD pathogenesis, these three macrophage subtypes were selected as phenotypic traits for the WGCNA. Outlier samples were identified in the GSE100153 dataset (Figure 1C) and removed (Figure 1D). The power was set to 5 when R^2^ > 0.8 (Figure 1E). A scale-free network was constructed, resulting in the identification of 30 gene modules (Figure 1F). Among these, three modules were significantly associated with the phenotypic traits (cor >0.3, P < 0.05) (Figure 1G).

### 3.2 Function of candidate genes in COPD

**FIGURE 2:**
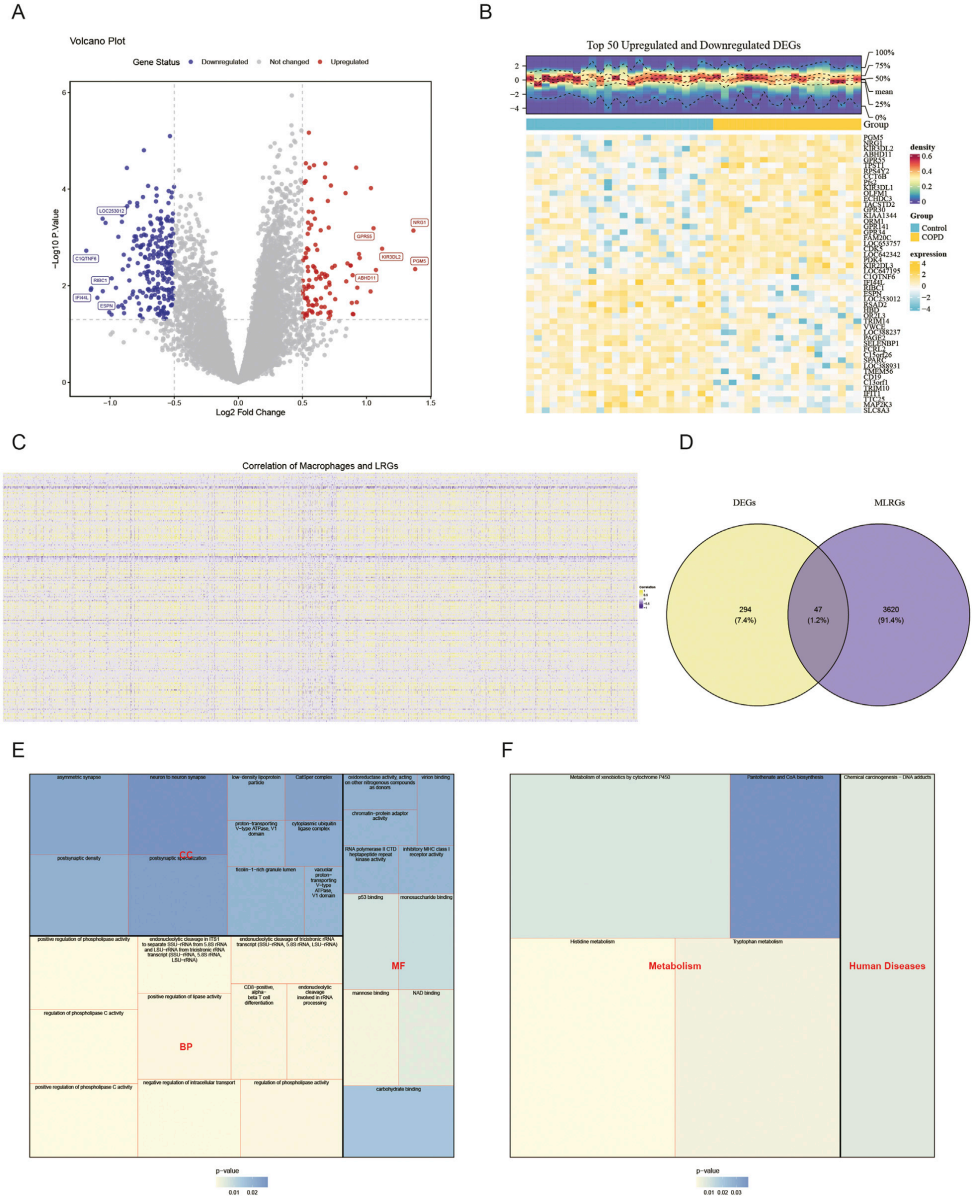
Function of candidate genes in COPD. In the GSE100153 dataset, the 106 up-regulated DEGs and 235 down-regulated DEGs (control vs. COPD) are shown in the volcano plot **(A)** and heatmap **(B)**. Red represents upward adjustment; blue represents downward adjustment. The 3,667 genes were defined as MLRGs by correlation analysis **(C)**. The Venn diagram in **(D)** shows that 47 DEGs and MLRGs overlap. GO **(E)** and KEGG **(F)** analyses of the candidate genes are shown.

Differential expression analysis revealed 341 DEGs in the COPD group compared to the control group, consisting of 106 up-regulated and 235 down-regulated genes. A volcano plot visualized all DEGs, with the top five up- and downregulated DEGs labeled based on their log_2_FC values (Figure 2A). A heatmap displayed the expression levels of the top 25 DEGs with the most significant up- and downregulation (Figure 2B). Correlation analysis identified 3,667 genes as MLRGs (Figure 2C). By intersecting the DEGs with MLRGs, 47 candidate genes were obtained (Figure 2D). These candidate genes were enriched in 390 functional categories, including 315 biological process (BP) terms (e.g., positive regulation of phospholipase C activity), 30 cellular component (CC) terms (e.g., ficolin-1-rich granule lumen), and 45 molecular function (MF) terms (e.g., mannose binding) (P < 0.05) (Figure 2E; Supplementary Table S3). KEGG pathway analysis indicated that the candidate genes were significantly enriched in 5 pathways, including histidine metabolism (P < 0.05) (Figure 2F). These results suggest that candidate genes may be involved in phospholipase regulation, molecular binding, or metabolic processes.

### 3.3 Three biomarkers in COPD

**FIGURE 3:**
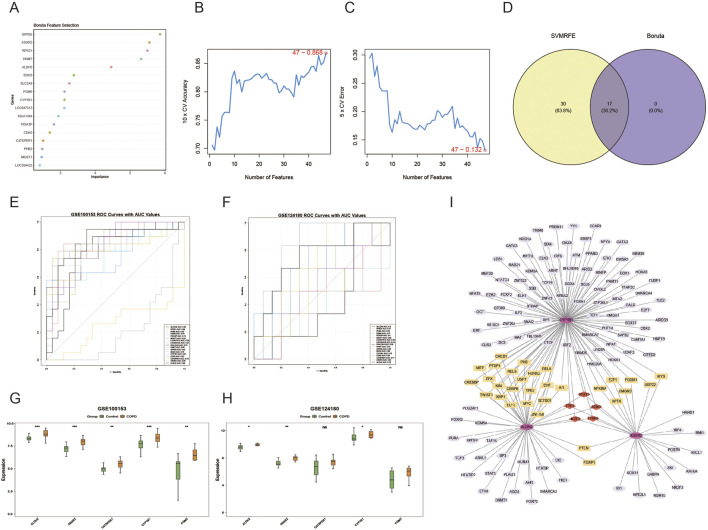
Identification of biomarkers in COPD. The 17 genes identified by the Boruta algorithm are shown in **(A)**, and the 47 genes identified by the SVM-RFE algorithm are shown in **(B,C)**. The 17 overlapping genes, obtained from the intersection of the two algorithm-based screening methods, are shown in **(D)**. Five genes (ALDH2, ASGR2, CYP1B1, CATSPER1, GM5) had AUC values greater than 0.7 in both GSE100153 **(E)** and GSE124180 **(F)**. Among these genes, only ALDH2, ASGR2, and CYP1B1 exhibited significant distinctions and consistent expression trends (control vs. COPD) (P < 0.05), in both GSE100153 **(G)** and GSE124180 **(H)**, *p < 0.05, **p < 0.01, ***p < 0.001. The transcription factor (TF) regulatory network for key genes is shown in **(I)**.

The Boruta algorithm identified 17 key genes (Figure 3A), and the SVM-RFE algorithm selected 47 genes (Figures 3B,C). By intersecting these two sets of genes, 17 candidate genes were subjected to ROC analysis (Figure 3D). The AUC analysis data were provided in Supplementary Table S4. Of these, five genes showed AUC values greater than 0.7 in both the GSE100153 (Figure 3E) and GSE124180 (Figure 3F) datasets. These genes were selected for further analysis. Notably, ALDH2, ASGR2, and CYP1B1 showed significant differences and consistent expression trends between COPD and control groups in both GSE100153 (Figure 3G) and GSE124180 (Figure 3H) datasets (P < 0.05). The expression levels of ALDH2, ASGR2, and CYP1B1 were significantly higher in patients with COPD compared to controls (P < 0.05). Therefore, ALDH2, ASGR2, and CYP1B1 were identified as the biomarkers for this study. Further analysis revealed that 154 TFs were associated with the biomarkers, with 5 TFs simultaneously targeting all three biomarkers, including STAT3 and SOX9 (Figure 3I). The regulatory relationships between biomarkers and TFs were visualized in a regulatory network, highlighting, for example, that ASGR2 and CYP1B1 are targeted by MYB.

### 3.4 Ability of biomarkers to predict COPD mortality

**FIGURE 4:**
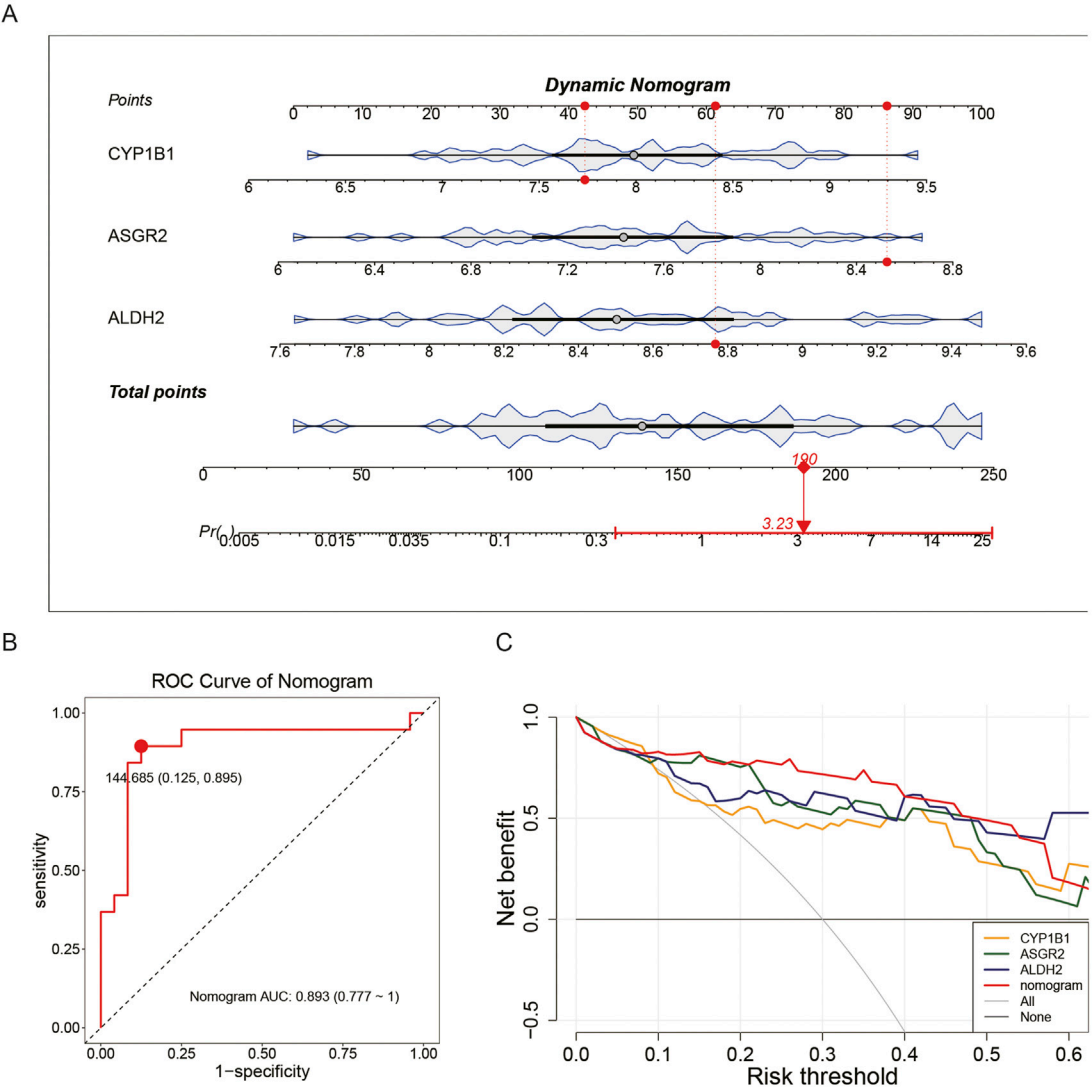
Ability of biomarkers to predict COPD mortality. In the GSE100153 dataset, a nomogram model was constructed based on biomarkers **(A)**. The AUC = 0.893 indicates the accuracy of the nomogram model **(B)**. The net benefit of the nomogram model further confirms its accuracy **(C)**.

A nomogram model based on the identified biomarkers was developed (Figure 4A). This model demonstrated its ability to predict the risk for patients with COPD, with the mortality rate increasing in correlation with the total score from the nomogram. An AUC of 0.893 confirmed the model’s accuracy (Figure 4B), while the maximum net benefit further validated its reliability (Figure 4C). The performance metrics of the model are presented in Supplementary Table S5. Overall, the biomarkers effectively predicted the mortality of patients with COPD.

### 3.5 Enrichment pathway of biomarkers

**FIGURE 5:**
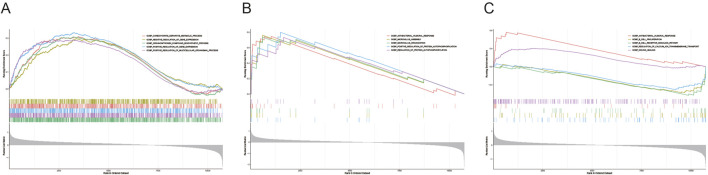
Enrichment pathways of biomarkers. GSEA was applied to explore the pathways enriched by CYP1B1 **(A)**, ALDH2 **(B)**, and ASGR2 **(C)**.

GSEA analysis revealed that CYP1B1 was enriched in 359 pathways, including translation, nucleic acid catabolic processes, and RNA processing (Figure 5A; Supplementary Tables S4, S6). ALDH2 was involved in 517 pathways, such as microvillus assembly and antibacterial humoral responses (Figure 5B; Supplementary Table S7). ASGR2 was associated with 32 pathways, including B cell receptor signaling, wound healing, and antibacterial humoral responses (Figure 5C; Supplementary Table S8). These results suggest that the biomarkers are linked to metabolic processes, B cell receptor pathways, and antibacterial humoral immune responses.

### 3.6 Ability of compounds to bind to biomarkers

**FIGURE 6:**
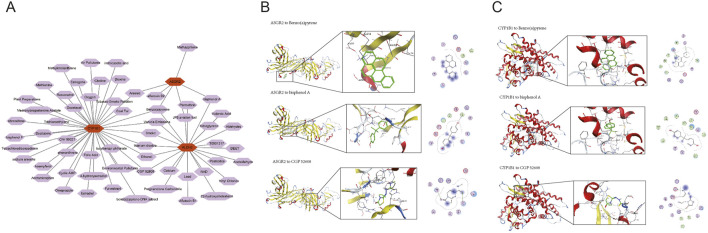
Ability of compounds to bind to biomarkers. The relationship network between biomarkers and compounds is shown **(A)**. ASGR2 exhibited strong binding affinity for CGP 52608, benzo(a)pyrene, and bisphenol A **(B)**. CYP1B1 exhibited strong binding affinity for benzo(a)pyrene, bisphenol A, and CGP 52608 **(C)**.

A database search identified 59 compounds related to the biomarkers. The relationship network revealed 8 compounds associated with multiple biomarkers, including arsenic (Figure 6A). Of these, protein structures for 5 compounds were available and subjected to molecular docking (Table 1). Notably, ASGR2 demonstrated a relatively strong binding affinity for CGP 52608 (Figure 6B). Additionally, ASGR2 exhibited binding capabilities with benzo(a)pyrene and bisphenol A (Figure 6B). CYP1B1 showed significant binding affinity for benzo(a)pyrene, bisphenol A, and CGP 52608 (Figure 6C), with the strongest binding energy observed for benzo(a)pyrene at −6.17870951 kcal/mol.

**TABLE 1:** Molecular docking results.

Biomarkers	PDB ID	Drugs	Compound CID	Binding energy (kcal/mol)
ALDH2	1NZX	Arsenic	5359596	—
		Benzo(a)pyrene	2336	—
		bisphenol A	6623	—
		CGP 52608	6509863	—
		Ethanol	702	—
ASGR2	8URF	Arsenic	5359596	—
		Benzo(a)pyrene	2336	−4.82352352
		bisphenol A	6623	−4.95880175
		CGP 52608	6509863	−5.11861801
		Ethanol	702	—
CYP1B1	3PM0	Arsenic	5359596	—
		Benzo(a)pyrene	2336	−6.17870951
		bisphenol A	6623	−5.83354139
		CGP 52608	6509863	−5.58286047
		Ethanol	702	—

### 3.7 Expression levels of biomarkers in clinical samples

**FIGURE 7:**
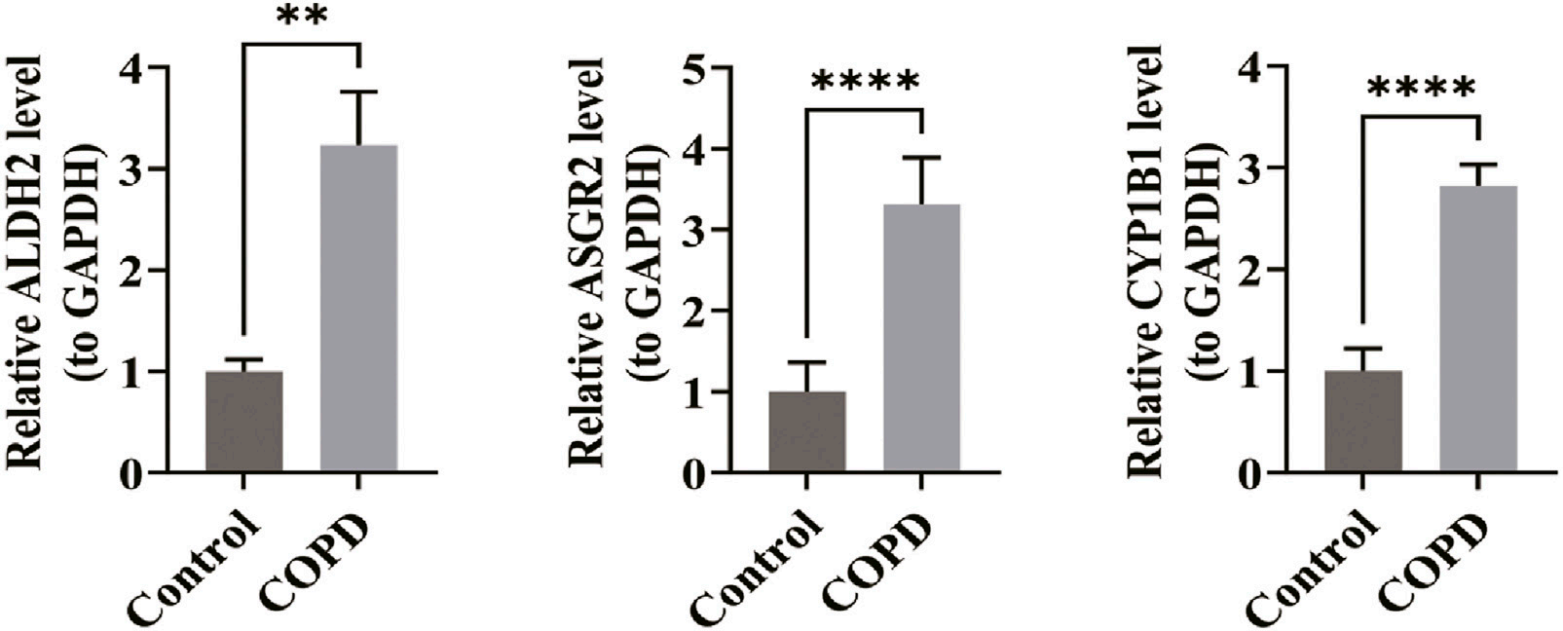
Expression levels of biomarkers in clinical samples. In the RT-qPCR experiment, the expression levels of ALDH2 (P < 0.01), ASGR2 (P < 0.0001), and CYP1B1 (P < 0.0001) were significantly higher in the COPD group compared to the control group (n = 5 per group). *p < 0.05, **p < 0.01, ***p < 0.001, ****p < 0.0001.

RT-qPCR analysis confirmed that the expression levels of ALDH2 (P < 0.01), ASGR2 (P < 0.0001), and CYP1B1 (P < 0.0001) were significantly higher in the COPD group compared to the control group (Figure 7). These findings aligned with the bioinformatics analysis results, reinforcing the validity of the conclusions drawn.

## 4 Discussion

COPD is a prevalent respiratory disorder and the fourth leading cause of global disease-related mortality, posing a significant economic burden worldwide (Brassington et al., 2022; Safiri et al., 2022). Given its profound impact, early detection is critical to slowing disease progression and alleviating pressures on healthcare systems. Emerging research underscores the close association between immune cell infiltration and COPD pathogenesis. In particular, macrophage polarization and lactylation—a recently identified post-translational modification—play central roles in immune dysregulation during COPD progression (Cruz et al., 2019; Bu et al., 2020). Thus, exploring the immune microenvironment, especially lactylation-related mechanisms, could provide novel diagnostic biomarkers that enhance clinical management and personalized therapies (Huang et al., 2022; MacDonald et al., 2023). In the present study, the infiltration levels of nine immune cell types showed significant differences between COPD and control groups, particularly among macrophages, including both M1 and M2 phenotypes. Additionally, three biomarkers associated with macrophage lactylation—ALDH2, ASGR2, and CYP1B1—were identified through bioinformatics analysis and further validated with clinical samples. GSEA analysis suggested that these biomarkers are primarily associated with metabolic pathways and B-cell receptor signaling. Furthermore, five TFs, including STAT3 and SOX9, were found to be linked to all three biomarkers. This study aims to identify significant novel biomarkers related to macrophage lactylation, which may aid in the early diagnosis and therapeutic management of COPD.

COPD is a chronic airway inflammatory disorder involving the participation of various immune cells and inflammatory mediators. Among them, macrophages, as the first line of defense in innate immunity, are one of the earliest immune cells to respond to inflammation. In COPD, macrophages play a key pathophysiological role via distinct M1 and M2 phenotypes (Lee et al., 2021). M1 macrophages, linked to infections or smoking-induced lung damage, promote Th1-type inflammation through pro-inflammatory cytokines (e.g., IFN-γ, TNF-α), exacerbating inflammation and tissue damage. In contrast, M2 macrophages, active in non-inflammatory states, aid tissue repair/remodeling and maintain homeostasis by secreting anti-inflammatory cytokines and clearing debris. Lactylation is a post-translational modification that adds lactate groups to proteins, thereby altering their function and stability. Recent study indicates that lactylation plays essential roles in regulating the M1/M2 polarization of macrophages (Bao et al., 2025). Research has showed that bone marrow-derived macrophages (BMDMs) stimulated with LPS and IFN-γ exhibited an M1 phenotype and increased lactylation. Following 24–48 h of M1 polarization, they began expressing M2-like genes (e.g., ARG1, VEGFA). In contrast, BMDMs treated solely with lactate displayed an M2-like phenotype and elevated lactylation without an initial M1 phase. These results indicate that lactylation induces an M2-like phenotype and facilitates the return of BMDMs to homeostasis during the late stage of M1 polarization (Zhang et al., 2019). However, the precise regulatory effects of lactylation on macrophage function have not been fully elucidated and need further investigation.

Through the application of machine learning algorithms and subsequent RT-qPCR validation of clinical samples, three diagnostic biomarkers associated with macrophage lactylation in COPD were identified. Aldehyde dehydrogenase 2 (ALDH2), a key enzyme involved in the metabolism of acetaldehyde, a product of alcohol metabolism, was one of these biomarkers (Wang et al., 2020). This tetrameric allosteric enzyme is highly expressed in critical organs such as the heart, brain, liver, and lungs (Ma et al., 2011; Koppaka et al., 2012). Reduced enzyme activity is associated with increased susceptibility to various diseases, including coronary heart disease (Xu et al., 2011), late-onset Alzheimer’s disease (Zhu Z. Y. et al., 2022), and cancer (Zhang and Fu, 2021; Tran et al., 2023). One study demonstrated that upregulating endogenous ALDH2 expression in fibrotic cells using CRISPR activation effectively inhibited the expression of profibrotic genes (Tan et al., 2021). Moreover, the ALDH2 loss-of-function polymorphism is linked to subtle alterations in pulmonary tissues, some of which resemble changes seen in normal pulmonary aging, suggesting a “premature lung aging” effect (Kuroda et al., 2017). A recent study has identified ALDH2 as a critical pathogenic mechanism linked to endogenous lactate accumulation in acute kidney injury and proposes it as a potential therapeutic target. ALDH2 lactylation at lysine 52 promotes PHB2 degradation via the ubiquitin-proteasome system, thereby inhibiting PHB2-mediated mitophagy and exacerbating mitochondrial dysfunction (Li J. et al., 2025). However, ALDH2 has not been studied in COPD. In this study, ALDH2 was significantly upregulated in COPD patients and may be involved in macrophage lactylation in the development of COPD, which might be helpful in the diagnosis and treatment of COPD.

Asialoglycoprotein Receptor 2 (ASGR2), a subunit of the asialoglycoprotein receptor, is a transmembrane protein predominantly expressed in hepatocytes. It specifically recognizes N-acetylgalactosamine and galactose and functions primarily in the internalization and degradation of glycoproteins via desialylation, a process essential for maintaining serum glycoprotein homeostasis (Grewal, 2010). ASGR2 expression correlates significantly with the clinical stage of hepatocellular carcinoma (Zhang et al., 2021). In gastric cancer, ASGR2 contributes to the manifestation of cancer hallmarks upon PS exposure and confers resistance to both chemotherapy and monoclonal antibody-based therapies (Kim et al., 2022). A recent study indicated that serum ASGR2 levels could serve as a biomarker for assessing the therapeutic effects of balloon pulmonary angioplasty (BPA) in patients with chronic thromboembolic pulmonary hypertension (CTEPH). Prior to BPA, ASGR2 levels were associated with HDL-C levels and platelet counts. Post-BPA, ASGR2 levels correlated with LYM%, which may provide insights into the immune and inflammatory states of patients with CTEPH (Xu et al., 2024). However, the specific regulatory mechanism of ASGR2 in COPD remains unclear and requires further in-depth exploration.

Cytochrome P450 Family 1 Subfamily B Member 1 (CYP1B1), a member of the CYP450 enzyme family, is expressed in both hepatic and extrahepatic tissues and plays a critical role in metabolizing a broad range of xenobiotics, including the metabolic activation of polycyclic aromatic hydrocarbons. CYP1B1 has been implicated in processes such as metabolism, inflammation, angiogenesis, and anticancer drug resistance (Li et al., 2017). CYP1B1 contributes to colorectal cancer (CRC) resistance to ferroptosis, with its metabolite, 20-HETE, mediating this resistance (Chen et al., 2023). Furthermore, CYP1B1 significantly influences CRC liver metastasis by regulating tumor cell proliferation through the “CYP1B1-LCFAs-G1/S transition,” suggesting its potential as a therapeutic target for CRC liver metastasis (Jin et al., 2023). It was reported that long-term exposure to incense smoke induces CYP1A1, CYP1A2, and CYP1B1 in rat lung and liver tissues with tissue-specific differences, accompanied by increased oxidative stress (elevated MDA and GSH levels, altered catalase activity in the liver) and inflammation (increased TNF-α and IL-4 levels), thereby potentially promoting carcinogenesis and health complications in chronically exposed individuals (Hussain et al., 2014). And CYP1B1 has been shown to play a protective role in preventing the exacerbation of allergic airway inflammation by ragweed extract and house dust mite, including increased IgE levels, infiltration of inflammatory cells, and especially an increase in Th2 cells (Alessandrini et al., 2022). In summary, these genes—ALDH2, ASGR2, and CYP1B1—are involved in key processes such as metabolism and inflammation. However, their expression and roles in COPD remain unexplored and need further exploration.

Previous studies have highlighted significant changes in the cellular components of the small airways in patients with COPD, with key alterations including epithelial cell senescence (Wu et al., 2022), a notable increase in neutrophils (Kapellos et al., 2023), elevated macrophage numbers accompanied by phenotypic shifts and impaired phagocytic function (Eltboli et al., 2014; Eapen et al., 2017), augmented T and B cell populations, and the proliferation and activation of epithelial dendritic cells (Stoll et al., 2015). In COPD, dysregulated immune cell activation and the release of immune mediators contribute significantly to the exacerbation of pulmonary inflammation. The altered immune microenvironment in COPD has gained increasing attention, and targeting immune cells could offer potential avenues for precision therapy (Tzortzaki et al., 2013; Lan et al., 2025). In the present study, immune infiltration analysis of MRGs revealed significant differences in the infiltration levels of nine immune cell types between the COPD and control groups, particularly monocytes, macrophages, dendritic cells, B cells, and CD8^+^ T cells. Macrophages, as integral components of the innate immune system, displayed significant differences between M1 and M2 phenotypes. Macrophage dysregulation is closely associated with COPD pathology and severity. Thus, strategies aimed at restoring the macrophage phenotype, improving phagocytosis, reducing inflammation, and addressing foamy macrophages may provide promising therapeutic targets for COPD. Furthermore, the role of innate immunity in COPD pathogenesis is complex, with different macrophage subsets contributing to proinflammatory responses, while M2 macrophages are involved in attenuating inflammation, promoting tissue repair, and decreasing the secretion of proinflammatory cytokines (Kim et al., 2024). Further analysis of the core genes identified their primary enrichment in metabolic pathways, B-cell receptor signaling, wound healing, and antibacterial humoral immune responses. Previous research has suggested that B cells, through antibody production, may play a role in airway inflammation in patients with COPD. B lymphocytes infiltrate the adventitia of small airways in patients with COPD, with a rise in lymphocyte fractions and lymphoid aggregates containing germinal centers as the disease progresses (Caramori et al., 2016). Previous research has indicated the antioxidant and wound-healing properties of baru nut extract in lung epithelial cells, suggesting its potential for COPD treatment (Coco et al., 2022). These findings offer valuable insights for future treatment strategies and further mechanistic investigations into COPD.

Database-based predictions identified 154 TFs associated with the biomarkers, with five TFs—STAT3, SOX9, TFAP4, IKZF2, and TP63—targeting all biomarkers. As is well established, the chronic inflammatory response plays a central role in the pathogenesis and progression of COPD. The JAK-STAT signaling pathway is crucial in the activation of cytokines during inflammation, significantly contributing to COPD development (Purohit et al., 2023). SOX9 alleviates cigarette smoke extract (CSE)-induced inflammatory injury in human bronchial epithelial cells by suppressing stromal interaction molecule 1 (STIM1) expression (Zhu X. et al., 2022). TFAP4, predominantly recognized as an oncogene (Wong et al., 2021), has recently been implicated in exacerbating liver fibrosis and tissue inflammation in mice by promoting the activation of the STING signaling pathway (Han et al., 2025). Moreover, the predicted compounds targeting the biomarkers primarily included CGP 52608, benzo(a)pyrene, and bisphenol A. These compounds warrant further exploration and may serve as the basis for the development of new targeted drugs, offering novel treatment options for COPD.

The PCR validation results provide strong evidence that the expression levels of biomarkers such as ALDH2, ASGR2, and CYP1B1 are significantly higher in patients with COPD. These findings, confirmed by clinical samples, align with the bioinformatics analysis and validate the reliability of the results. Consequently, this study offers clinical value for diagnosing COPD and identifying novel potential immune targets for COPD immunotherapy.

However, this study still has some limitations. Firstly, the clinical sample size of this study is relatively small, which may compromise the generalizability of the conclusions. Secondly, the samples validated via RT-qPCR in this study were whole blood, rather than those of purified macrophages or lung tissues, and thus may be confounded by the influence of other blood cell types. Thirdly, alterations in mRNA levels can be affected by multiple factors; relying solely on such changes is insufficient to definitively elucidate the lactylation status. There is a paucity of experimental validation and biological functional verification that directly link these biomarkers (ALDH2, ASGR2, and CYP1B1) to lactylation, coupled with potential batch effects in public databases (e.g., data from acute exacerbation phases, which fail to fully capture the heterogeneous features of chronic obstructive pulmonary disease). In addition, the data acquisition technologies used for the training set and validation set in this study are different (microarray technology and RNA sequencing). Such a difference may cause some DEGs to fail to be validated across platforms, which may further affect the completeness of candidate gene screening. In future studies, we will perform additional experiments to validate the findings. Firstly, we will employ macrophage-specific gene knockout models or organoid technology to mechanistically dissect how ALDH2/ASGR2/CYP1B1 regulates cell polarization through lactylation. Secondly, we will collect a more diverse set of samples (e.g., macrophages or lung tissues) for RT-qPCR or proteomic assays to validate changes in protein levels and their functional implications, thereby mitigating the influence of other blood cells. Thirdly, we will expand the scale of the dataset, attempt to use data from the same technical platform to reduce the impact of technical bias on the results, and strive to evaluate the relationship between biomarkers and disease stages—especially those biomarkers associated with metabolic syndrome in COPD. Additionally, we will incorporate mass spectrometry (MS) analyses to detect whether these proteins undergo lactylation under specific conditions, and validate the lactylation status of relevant enzymes or target proteins via Western blotting using anti-lactylation-specific antibodies, so as to more precisely characterize the modification status of proteins. Furthermore, leveraging the strong interaction between CYP1B1 and benzo[a]pyrene, we will develop targeted inhibitors or environmental exposure interventions. We aim to fill existing gaps through these studies and lay the groundwork for an in-depth understanding of the immunometabolic mechanisms of chronic obstructive pulmonary disease and their translational applications.

## 5 Conclusion

In conclusion, this study identified ALDH2, ASGR2, and CYP1B1 as novel biomarkers related to macrophage lactylation in COPD, demonstrating their roles in regulating the immune microenvironment (e.g., macrophage polarization) and metabolic pathways (e.g., oxidative stress, B-cell signaling). These findings provide new targets for early diagnosis and therapy.

## Data Availability

The datasets (ANALYZED) for this study can be found in the [GEO database (GSE100153 and GSE124180)] (https://www.ncbi.nlm.nih.gov/geo/). If original data were required, the corresponding authors was contacted. We are glad to provide the original data or add them to the attachment.

## Supplementary material

SUPPLEMENTARY TABLE 1 The lactylation-related genes.

SUPPLEMENTARY TABLE 2 The experimental conditions of RT-qPCR.

SUPPLEMENTARY TABLE 3 The functions of 47 candidate genes.

SUPPLEMENTARY TABLE 4 CYP1B1 was enriched in nucleic acid catabolic processes.

SUPPLEMENTARY TABLE 5 The performance metrics of the model.

SUPPLEMENTARY TABLE 6 CYP1B1 was enriched in RNA processing.

SUPPLEMENTARY TABLE 7 Enrichment pathway of ALDH2.

SUPPLEMENTARY TABLE 8 Enrichment pathway of ASGR2.

## Abbreviations

COPD, Chronic obstructive pulmonary disease; CT, Computed tomography; TRMs, Tissue-resident macrophages; DEGs, Differentially expressed genes; WGCNA, Weighted gene co-expression network analysis; GSEA, Gene Set Enrichment Analysis; GEO, Gene Expression Omnibus; LRGs, Lactylation-related genes; MRGs, Macrophage-related genes; MLRGs, Macrophage lactylation-related genes; GO, Gene Ontology; KEGG, Kyoto Encyclopedia of Genes and Genomes; ROC, Receiver operating characteristic; SVM-RFE, Support Vector Machine-Recursive Feature Elimination; AUC, Area under curve; MSigDB, Molecular Signatures Database; TFs, Transcription factors; MOE, Molecular operating environment; RT-qPCR, Reverse transcription quantitative PCR; BP, Biological process; CC, Cellular components; MF, Molecular functions; BPA, Balloon pulmonary angioplasty; CTEPH, Chronic thromboembolic pulmonary hypertension; CRC, Colorectal cancer; CSE, Cigarette smoke extract.
